# Profound Context-Dependent Plasticity of Mitral Cell Responses in Olfactory Bulb 

**DOI:** 10.1371/journal.pbio.0060258

**Published:** 2008-10-28

**Authors:** Wilder Doucette, Diego Restrepo

**Affiliations:** Department of Cell and Developmental Biology, Neuroscience Program and Rocky Mountain Taste and Smell Center, University of Colorado Denver Anschutz Medical Campus, Aurora, Colorado, United States of America; University of Maryland, United States of America

## Abstract

On the basis of its primary circuit it has been postulated that the olfactory bulb (OB) is analogous to the retina in mammals. In retina, repeated exposure to the same visual stimulus results in a neural representation that remains relatively stable over time, even as the meaning of that stimulus to the animal changes. Stability of stimulus representation at early stages of processing allows for unbiased interpretation of incoming stimuli by higher order cortical centers. The alternative is that early stimulus representation is shaped by previously derived meaning, which could allow more efficient sampling of odor space providing a simplified yet biased interpretation of incoming stimuli. This study helps place the olfactory system on this continuum of subjective versus objective early sensory representation. Here we show that odor responses of the output cells of the OB, mitral cells, change transiently during a go–no-go odor discrimination task. The response changes occur in a manner that increases the ability of the circuit to convey information necessary to discriminate among closely related odors. Remarkably, a switch between which of the two odors is rewarded causes mitral cells to switch the polarity of their divergent responses. Taken together these results redefine the function of the OB as a transiently modifiable (active) filter, shaping early odor representations in behaviorally meaningful ways.

## Author Summary

The way in which the brain represents and processes sensory information remains a fundamental question. One model posits that stable neural representation of a stimulus during early stages of stimulus processing allows for unbiased interpretation of incoming stimuli by higher order cortical centers. Alternately, early stimulus representation could be shaped by previous experience, thus providing a biased yet relevant interpretation of incoming stimuli. This study examines the activity of output cells, mitral cells, from the first stage of odor information processing in the olfactory bulb during an odor discrimination task. We found that odor responses changed during the task in a manner that increased the ability of the circuit to convey information necessary to discriminate among closely related odors. A switch between which of the two odors were rewarded caused mitral cells to switch the polarity of their divergent responses in behaviorally relevant ways. These results show that early neural representations of odor can be shaped by previously derived meaning, providing a simplified yet biased interpretation of the odor environment to higher cortical structures.

## Abstract 3

Early neural representation of odor can be shaped by previously derived meaning, providing a simplified yet biased interpretation of the odor environment to higher cortical structures.

## Introduction

Olfactory sensory neurons (OSNs) express one of hundreds of olfactory receptors encoded in the genome, and all neurons expressing the same receptor target their axons to one or two ovoid neuropil structures called glomeruli at the surface of the olfactory bulb (OB) [1,2]. Within the glomeruli axons from olfactory sensory neurons responsive to odorant features recognized by the particular receptor they express make synapses onto the primary dendrites of mitral and tufted cells, the output cells of the OB. Thus, glomeruli are functional units of activity, analogous to cortical barrels or columns, and odors appear to be encoded by a combinatorial code of glomeruli activated by odors [3,4]. These maps of activity at the glomerular layer of the bulb contain enough information to differentiate between odors and undergo variations in time that may contribute to the information conveyed to the brain. However, the use of this information poses a challenging problem for the brain because of the large number of glomeruli activated by each odor, and the high degree of overlap in the glomerular activity patterns of closely related odors [5–9].

The OB is the first relay station in the brain where incoming information on odors is processed. Mitral and tufted cell activity is modified by a large population of interneurons [4,10–12] that includes the periglomerular cells with cell bodies located surrounding the glomeruli and the granule cells that form dendro-dendritic reciprocal synapses with lateral dendrites of mitral and tufted cells. Of these interneurons the granule cells are known to influence information transmission to the olfactory cortex by activity-dependent lateral inhibition, oscillatory coupling, and cell pair synchronization of mitral cells (MCs) [13–15]. Lateral interactions mediated by interneurons result in changes in odor representation by MCs that evolve as a function of time during the response to an odor [16–19]. In this respect, this circuit resembles processing in the retina. However, there are large differences between mammalian retina and the OB: First, the input to the OB appears to be actively filtered by modulation of how the environment is sampled through sniffing [20,21]. And second, the OB has massive centrifugal innervation that is thought to modulate the interaction of interneurons and mitral/tufted cells [4]. This centrifugal innervation includes noradrenergic, serotonergic, and cholinergic fibers as well as feedback from olfactory cortex and anterior olfactory nucleus (AON). Numerous studies have shown that this centrifugal innervation has the ability to modify MC responses [22–25].

Modulation of the circuitry in the main OB by centrifugal innervation in vertebrates [26,27] and the antennal lobe in insects [28,29] occurs during learning to discriminate different odors and these changes in circuit processing are in part dependent on noradrenaline in vertebrates [30] and octopamine in insects [31]. In a review of the literature on olfactory learning, Davis postulated that these changes could represent either an increase in the number of neurons responding to an odor or an increase in synchronized neuronal firing [32], but the evidence available to validate this postulate in vertebrates remains inconclusive.

The only study that recorded changes in odor responsiveness in single MC units during learning in awake behaving vertebrates is that of Kay and Laurent [33]. These investigators found that in vivo MC odor responses were not as robust as reported in previous studies in anesthetized animals and were only weakly modulated by respiration, a premise that has been substantiated by Rinberg and coworkers [34] and Davison and Katz [35] who find sparse odor responses in awake behaving animals. Importantly the studies by Kay and Laurent suggested that MC activity was modulated by behavioral context. Unfortunately the paucity of the data (only six units showed odor-selective firing rates with small signal-to-noise ratios) precluded determination of whether the responses were a reflection of behavioral actions associated with the odors or a response to the odors themselves. In contrast, studies with multielectrode arrays or optical imaging of activity of projection neurons in insect antennal lobe showed that individual cells responding to learned odors change their response profile during learning [28,29], raising the question whether similar changes occur in vertebrates.

Here we describe experiments where the use of multielectrode implantation in the main OB of mice enabled us to thoroughly characterize changes in the activity of neuron ensembles in the MC layer of the OB during odor discrimination learning. We find transient development of divergence in the firing of MCs between reinforced and unreinforced odors during an odor discrimination learning task. Strikingly, odor responses change when the odor associated with reward is switched, implying profound context-dependent plasticity in the representation of odors by MCs.

## Results

### Multiple Odor Screening and Multielectrode Array Recording Allow Measurement of Sparse Odor Responses

**Figure 1:**
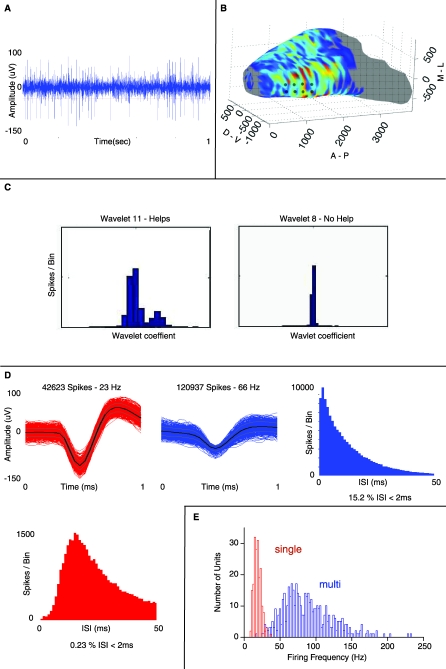
Raw Data, Electrode Location, and Spike Sorting (A) One second of raw data collected from a single electrode and filtered at 300–3,000 Hz. (B) Ventral view of a 3D reconstruction of the OB with benzaldehyde activation pattern [73] illustrating the approximate location and layout of the tips of the electrode array (black dots). (C) Wavelet coefficient that was (Helps) used (left) and not used (No Help) (right) for sorting of spikes thresholded from the channel shown in (A). Waveform descriptors, like wavelet coefficients, that create non-normal distributions of spikes are effective for grouping spikes into clusters. (D) Sorted waveforms displayed with corresponding ISI histograms. The red unit was classified as a single unit because it displayed less than 3% of total spikes violating an ISI less than 2 ms (see Materials and Methods). (E) Histogram showing the distribution of prestimulus firing frequencies for 660 units (189 single units and 471 multi units). The red histogram is the distribution for the single units (18.5 ± 5 Hz, *n* = 189, mean ± SD), and the blue histogram is the distribution for the multi units (94 ± 33 Hz, *n* = 471).

Mice were implanted bilaterally with 2 × 4 multielectrode arrays with tips targeted to the ventral MC layer in the OB (Figure 1B) to allow for determination of how MC activity changed during learning in an olfactory discrimination task. Each of the 3–4-MΩ Pt/Ir electrodes in the array was tapered to an exposed tip of 2 μm. These electrodes did not detect voltage fluctuations above three times the standard deviation (SD) of the noise (3 × SD) in the granule cell layer, but recorded many events where the voltage deviated from baseline by more than 3 × SD when the electrode tips crossed the MC layer during surgical implantation of the chronic electrodes. Figure 1A illustrates a raw trace from a single electrode with tips placed in the MC layer. Spikes were detected as those voltage deviations that exceeded 3 × SD, and units were sorted using the program Wave_Clus (with slight modifications, see Materials and Methods) that employs superparamagnetic clustering of the coefficients resulting from a wavelet transform of the spike waveforms (Figure 1C) [36]. Figure 1D shows examples of two sorted spikes isolated from the same electrode as well as the interspike histograms corresponding to each of these spikes. Spikes with less than 3% of the interspike intervals (ISIs) in the refractory period (<2 ms) were designated as single units (red in Figure 1D), and all other spike groups were designated as multiunits (blue in Figure 1D). Using this approach, the multielectrode arrays allowed us to record suspected mitral cell (SMC) activity from an average of 12 multiunits and five single units per experiment. As expected from recordings from MCs, these units fired in robust respiratory bursts when recorded under anesthesia (unpublished data) and the basal firing rate for single units varied from 8–36 Hz (18.5 ± 5 Hz, mean ± SD, *n* = 193, see Figure 1E). Because our electrodes are not able to record from granule cells and because respiratory bursts in anesthetized animals and the basal firing rates are typical of MCs [33,34], we refer to these units in the rest of the manuscript as suspected mitral cells (SMCs).

**Figure 2:**
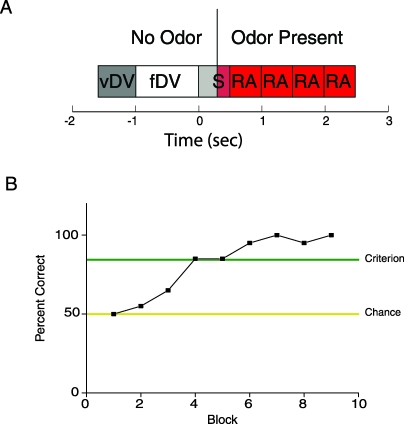
Go–No-Go Odor Discrimination Task. (A) Time course for each trial in the odor discrimination task. The trial is started by a nose poke of the mouse into the odor chamber. When the computer senses the nose poke it turns a valve that diverts the air flow of 2 l/min to the exhaust and it turns on the odor valve that injects odorized air into the main air flow at 40 ml/min. The mouse must remain in the chamber for a time period made up of a variable 0–0.5-s period (variable diverting valve period or vDV) followed by a fixed 1-s interval (fDV). At the end of the fixed diverting valve (fDV) period the odor has mixed thoroughly with the main air flow, and the diverting valve directs the air flow back into the chamber (time 0 s). At this time the mouse must stay for 0.5 s in the chamber (S) and then must lick at least once in each of the 0.5-s response area (RA) segments if the odor is a rewarded odor. If the mouse licks in the four RA segments, the mouse is rewarded with water flowing through the tube it has been licking. A 6-s time out follows the end of the trial. If the odor is an unrewarded odor the mouse does not have to lick, and typically withdraws the head from the odor port shortly after it makes a decision. While the diverting valve is activated at time zero, there is a delay in delivery of the odor that we estimate to be of the order of ∼300 ms. (B) Typical curve for behavioral performance in an odor discrimination session. The percent correct response is shown as a function of block number. Each block includes ten rewarded odor and ten unrewarded odor trials. A correct response is licking of the tube in the four RA segments for a rewarded odor and not licking in at least one RA period for the unrewarded odor (this is the go–no-go criterion). Note that this mouse starts with chance performance (50%) and reaches the arbitrary response criterion of 85% correct by four blocks.

We sought to determine whether SMC odor responses would change as the animal learned to discriminate between two odors. We trained mice to perform a go–no-go task in which thirsty animals were rewarded with water when they licked on a response tube in the presence of the correct odor in the pair being tested (described in detail in the Materials and Methods) [37]. Figure 2A shows the events associated with each trial. The trial was initiated by the mouse by poking its nose into an odor port. This initiated a sequence of events. First, the air stream was diverted away from the odor sampling port to an exhaust port by a valve, and the mouse had to wait with its head inside the odor port for a period when odor was not present in the chamber. This diverting flow interval varied randomly from 1 to 1.5 s (this is the interval denoted by vDV + fDV in Figure 2A). After the end of the diverting flow interval the valve shifted the air flow back into the odor port, but this time the air stream carried the odor. The instant when the diverting valve shifts the air stream back into the odor port is time zero in all our trials. Odor reaches the odor sampling chamber a fraction of a second later (we estimate ∼0.3 s, but this time would have to be measured to determine a precise delivery time). Mice were asked to respond to the rewarded (S+) odor by licking on a tube at least once in each of four 0.5-s intervals that span the time from 0.5 to 2.5 s (the response area in Figure 2A). Licking at least once in each of the response area intervals is the go–no-go criterion. Note that the mouse is not required to lick during the unrewarded odor (S- trial) and therefore the mouse is free to leave the port once it makes a decision.

Figure 2B shows percent correct responses for a mouse in a go–no-go session in which the mouse was asked to respond to isoamyl acetate as the rewarded (S+) odor and refrain from licking the tube when the unrewarded (S−) odor (cumin aldehyde) was presented. Each block included ten trials with the S+ odor and ten with the S− (S+ and S− trials were interspersed pseudorandomly). As shown, the mice typically attained criterion (arbitrarily set at 85%) after four blocks of 20 trials. Because we needed to use odors that would result in responses in SMCs whose activity was being monitored by at least one electrode in the array, we had to screen multiple odors before starting an odor discrimination session. As described in detail in the Materials and Methods section we attained this by screening the SMCs for responsiveness to a subset of 12 odors on day 1, processing the data overnight, and then performing a go–no-go odor discrimination task with responsive odors on day 2. Odor responsiveness screening on day 1 occurred while the mouse was passively exposed to different odors (see Materials and Methods for a detailed description and odors used). For the day 2 go–no-go task the rewarded odor (S+) was chosen to be one of the odors that elicited responses on day 1 (odor A), and the unrewarded (S−) odor was a 1:1 mixture of the S+ odor and another odor that had elicited responses in day 1 (odor AB). Mice were subjected to day 1/day 2 sessions repeatedly over a 2-mo period (typically twice per week). The same odor was never used in two different day 2 sessions, thus, the mice were always learning to differentiate between novel odors. The combined use of microelectrode array recording and prescreening of multiple odors was used to allow us to record odor responses of SMCs during odor discrimination learning sessions.

### Prescreened Odors Elicit Frequent Responses in SMCs

**Figure 3:**
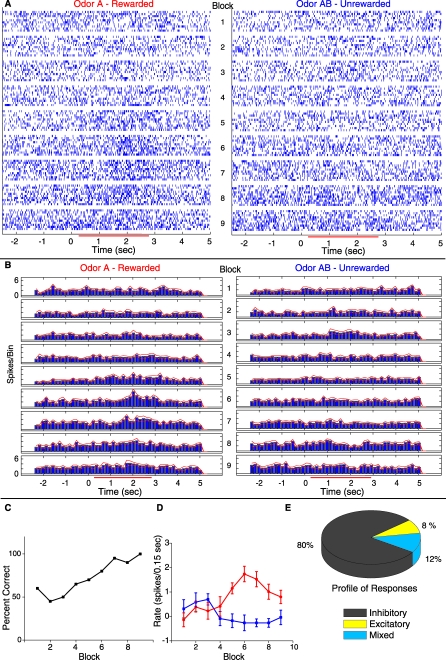
Divergence in Single Unit Responses during Learning in the Odor Discrimination Task (A) Raster plot of single unit spike times organized per block for the ten rewarded trials (A, left column) and ten unrewarded trials (AB, right column). Timing and duration of odor exposure is indicated on the *x*-axis by the red bar. (B) PSTH of the data shown in (A). Red lines on either side of the histogram indicate +/− standard error of the mean (SEM). The bin size in the PSTH is 0.15 s. This means that the firing rate in Hz is the value in the *y*-axis × (1/0.15). (C) Behavioral performance—percent correct as a function of block number—for the animal from whom the cell in (A) and (B) was recorded. (D) A plot of the firing-rate increase above background to odor A (red) and odor AB (blue) in each block of the behavior. The points represent the firing rate in spikes/0.15-s bin during odor exposure (0.5 to 2.5 s) minus the rate in spikes/0.15-s bin in the period immediately before odor exposure (−1 to 0 s). Error bars denote the mean +/− SEM of each point (ten trials per point). (E) The lower right hand pie chart shows what percent of the responses were inhibitory (gray), excitatory (yellow), or mixed (blue). A mixed response was defined as a response to either odor A or AB that had both an excitatory and inhibitory component or a response that was excitatory to one odor and inhibitory to the other odor stimulus.

Figure 3A displays a raster plot and Figure 3B shows the corresponding peristimulus histogram (PSTH) depicting the firing of a single unit during an olfactory discrimination task. The left panel shows activity during the rewarded odor trials (odor A) and the right panel shows activity of the SMC for the unrewarded odor (odor AB) trials. Each block of 20 trials (ten rewarded and ten unrewarded) is represented by the set of ten rows in the right panel (unrewarded) and the corresponding set of ten rows in the left panel (rewarded). The nine blocks that composed the learning session are displayed from top to bottom. Figure 3A illustrates how the odor response of one SMC evolves as the animal learns to discriminate between the two odors, developing what appears to be an excitatory response to the rewarded odor and no response to the unrewarded odor. In order to determine whether the firing rate during odor exposure (peristimulus interval) differed significantly from the firing rate before odor exposure (prestimulus interval), we used a *t*-test with correction for multiple comparisons to compare the difference between the firing rate in the prestimulus interval (−1 to 0 s) to the firing rate in small windows (0.75 s) scanned across the peristimulus interval (0.5 to 3.125 s) by 0.325-s steps (see Materials and Methods). The *p*-values of this test for significant responses for the SMC shown in Figure 3A are displayed in Table 1. The rewarded odor induced significant changes in firing rate in blocks 5, 6, and 7 and the unrewarded odor elicited a significant change in block 3.

**Table 1:**
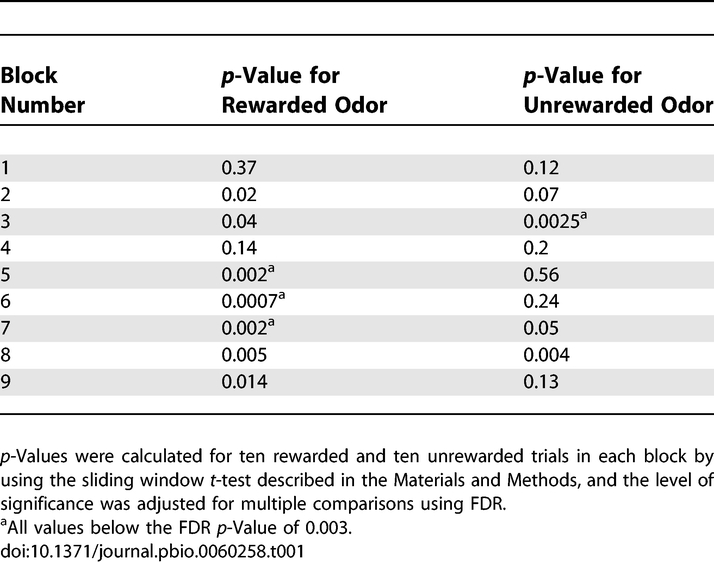
*p*-Values for Significance of the Difference between Prestimulus and Peristimulus Rate for the Data in Figure 3B

We proceeded to analyze all experiments using this statistical test. Among the 660 units (189 single units and 471 multi units) surveyed in a total of 38 odor discrimination sessions performed by eight animals in this study, 373 (56%) displayed a difference in firing rate between the post- and prestimulus intervals. Only 20% of the odor-responsive SMCs responded with excitation to one of the two odors during the peristimulus interval (as shown for the unit in Figure 3A and 3B) while 80% displayed only reduced firing rate responses during the peristimulus interval (Figure 3E). Thus, the use of odors prescreened for their ability to elicit responses in the subset of SMCs that were being monitored by the multielectrode array resulted in frequent recording of putative odor responses during the odor discrimination task allowing us to study changes in odor responses systematically.

### MC Responses to the Rewarded and Unrewarded Odors Diverge During the Odor Discrimination Task

**Figure 4:**
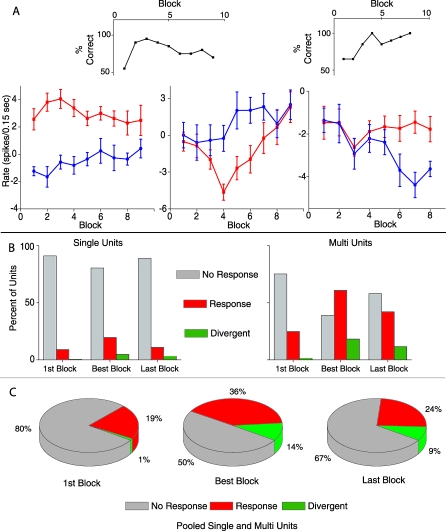
Transient Firing Changes Observed during Learning (A) The top black plots display the behavior of the animal from which the unit data displayed in the lower plots was recorded. The behavior plot on the left corresponds to the two plots in the lower left and the upper right behavioral plot goes with the lower right rate change plot. The lower plots show unit firing rate change in response to odor A (red/rewarded) and odor AB (blue/ unrewarded) in each block of the behavior. The points represent the firing rate in spikes/0.15-s bins during odor exposure (0.5 to 2.5 s) minus the rate in spikes/0.15-s bin in the period before odor exposure (−1 to 0 s) (for details see Materials and Methods). Error bars denote mean +/− SEM of each point. (B) Bar graphs depicting the percent of total units that were responsive(red) and divergent (green) in the first, best, and last blocks. The data are from 189 single units (left) and 471 multiunits (right) recorded from eight different animals in 38 sessions. (C) The three pie charts illustrate the percent of total units (pooled single and multiunits) that were responsive (red) and divergent (green) in the first, best, and last blocks. The data are from 660 units recorded from eight different animals and 38 separate odor discriminations.

In awake behaving animals there are MC responses to behavioral events such as poking the nose into the odor delivery chamber. As discussed in detail by Rinberg and coworkers [34], this fact makes it difficult to assign the changes in firing rate between pre- and peristimulus periods to odor responses unambiguously. Rinberg and coworkers propose detecting differences in firing rate between the rewarded and unrewarded odors in the peristimulus interval as a more robust method to determine odor responses. In this section we present comparisons of the difference in firing rate in a time window when the mouse is exposed to the odor as a robust measure of changes in firing rate caused by odor responses. The SMC whose firing is illustrated in the raster plots and peristimulus time histograms shown in Figure 3A and 3B appears to display a differential response to odors in blocks 5–7. The relationship of this cell's response divergence with the behavioral performance of the animal can be appreciated by comparing Figure 3A or 3B with Figure 3C where the percent of correct behavioral responses is plotted as a function of block number. Note that what we mean by response divergence is the difference in SMC firing rate during the peristimulus interval between rewarded odor trials and unrewarded odor trials computed on a block by block basis. It appears that the cell begins to diverge in its response to the unrewarded and rewarded odors at around block 5 while the animal does not begin performing at behavioral criterion (85% correct) until block 7. Indeed, when the changes in firing rates upon odor application (peristimulus minus prestimulus rates) are plotted as a function of block number for the reinforced and unreinforced odors (Figure 3D) the changes differ, not only across blocks, but also across stimuli (as shown by an ANOVA at *p* < 0.05). A post-hoc test shows that a statistically significant divergence in firing rate does in fact occur by block 5. Interestingly, the response is transient as evidenced by a lack of a statistical difference in a post hoc test between firing rates by the last block. Figure 4A shows other examples of the relationship of divergent firing between rewarded and unrewarded odor responses (all of these differed between stimuli when tested with ANOVA at *p* <0.05). The left plot of Figure 4A shows a unit that displayed differential firing from the beginning to the end of the session (very few units, two of 660, displayed differences throughout the session and this is shown here only for completeness). The center trace shows another unit from the same animal in which the unit develops a reduced firing rate response to the rewarded odor and no response—or perhaps a small excitatory response—to the unrewarded odor. The right plot in Figure 4A shows an example of a unit where the rewarded response is a stable decrease in the firing rate and the unrewarded response becomes a significantly larger decrease in firing rate than the rewarded response, developing into a divergent response.

The unit responses presented in Figures 3D and 4A appeared to diverge during the odor discrimination task. To assess whether this was a general phenomenon all units were analyzed for divergence in their response to the rewarded versus the unrewarded odor. In order to determine systematically which units differ in firing rate during odor exposure we compared the firing rates of reinforced and unreinforced trials using a *t*-test with correction for multiple comparisons in a window of 0.75 s that was slid along the peristimulus interval (see Materials and Methods for details). Units were classified as divergent if the firing rate in the 0.75-s window differed significantly between unrewarded and rewarded odors in at least two blocks. The percent of units that differ in firing rate between the rewarded and unrewarded trials is shown in Figure 4B and 4C. These data are calculated from recordings from 660 units surveyed (189 single units and 471 multi units) in a total of 38 odor discrimination sessions performed by eight animals. Results are shown for the first and last blocks in the session, as well as for the “best block” defined as the block where the firing rates differed most significantly (smallest *p*-value) for a given unit. The median best block was between the fifth and sixth block and the best block ranged from three to ten.

The green bars or slices of Figure 4B and 4C and the data in Table 2 illustrate how the percent of divergent SMCs change from the first block to the best block to the last block for all animal–odor-pair discriminations. Indeed a significant increase in the number of divergent units occurs between the first and the best block (Chi^2^ *p*-value < 0.05) and a moderate statistically significant drop off from the peak value is observed by the last block. Table 2 also shows that separate analysis of single- and multiunit activity yields qualitatively similar results. Because of this we pooled the single- and multiunit data in all subsequent analysis. As with divergent units, the number of responding units showed both a significant increase from first block to best block as well as a significant decrease from best block to last block (Chi^2^ *p*-value < 0.05) (Figure 4C, red pie). Unit responses were defined as either a significant increase or decrease in firing rate from background in response to either the rewarded or unrewarded odor. This transient increase in unit responses is largely made up of reduced firing rate responses (as opposed to excitatory responses) as would be expected by the large percent of reduced firing responses shown in Figure 3E.

**Table 2:**
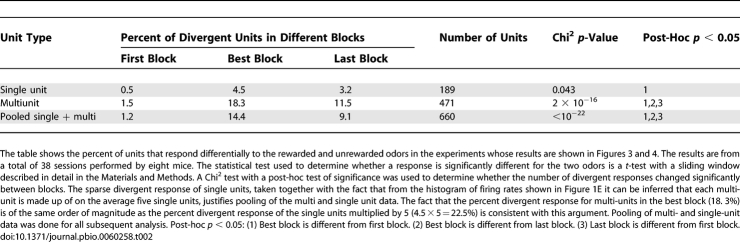
Percent of Units That Respond Divergently in the Odor Discrimination Task

In order to assess whether the statistical differences in firing rate between reinforced and unreinforced odors was real, this procedure was implemented during the prestimulus interval. If our analysis was stringent enough then running it in the prestimulus time period would not result in any significant divergence in firing between rewarded and unrewarded trials. When this analysis was performed only one out of 660 units tested had a significantly divergent firing rate compared with 95 of 660 units that were significantly divergent during the peristimulus interval. Thus, the statistical test used to determine whether the units respond differentially to the odors was found to be robust.

### Divergent Responses to the Reinforced and Unreinforced Odors Are Responses to the Odor Stimulus Rather Than to Behavioral Events Associated with Odor Presentation

A common confound in evaluating whether divergent neural activity is stimulus driven is the possible link between the response and the behavioral action as opposed to the expected relationship between the response and the stimulus [38]. Simply, are the observed SMC responses shown in Figures 3 and 4 really olfactory driven or do they merely represent planned/actual behavioral action, such as licking of the reward tube? This is particularly relevant in recordings in the OB where it is well known that actions such as nose poke elicit changes in MC firing [33,34]. To determine whether the divergent responses were odor driven, responses to a given odor were compared between trials in which the incorrect or correct behavioral action was observed. This was done for each unit in blocks where the firing rates differed significantly between the rewarded and unrewarded odors. Trials from these significantly divergent blocks in which the animal made the wrong behavioral response were used to compare with trials in which the correct behavioral action was observed.

**Figure 5:**
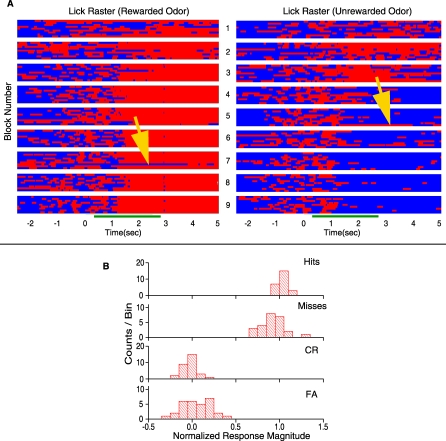
Lick and Odor Responses Where the Animal Made Correct or Incorrect Behavioral Responses (A) Trial by trial rasters of lick behavior for the rewarded (odor A, left) and unrewarded (odor AB, right) odors. Red indicates periods of licking and blue indicates periods of no licking. Data for ten trials are shown for rewarded and unrewarded odors per block. Blocks are arranged from top to bottom. The green bar below the rasters indicates when the odor was delivered to the chamber. The yellow arrows point to trials where the animal made a mistake in the lick response. (B) Histograms of response magnitude normalized to the average correct rewarded (1) and unrewarded (0) firing rates during the peristimulus period sorted for the four different types of behavioral outcomes. The normalized response magnitude was calculated for each unit for all divergent units in 11 sessions in four animals (these were a subset of the 34 sessions in Figure 4 where the animal made six or more mistakes). Hits are trials where the animal licks sufficiently to obtain a water reward during a rewarded odor trial. Misses are trials in which the animal fails to lick sufficiently to receive reward on rewarded odor trials. Correct rejections (CR) are trials in which the animal refrains from licking during an unrewarded odor trial. False alarms (FA) are trials in which the animal responds by licking to an unrewarded odor as if it were a rewarded trial. The number of counts per bin represents the number of units displaying a response of a given normalized magnitude.

Figure 5A illustrates the licking responses for all trials in the experiment whose data were presented in Figure 3A. For each trial the bin was assigned a value of 1 (red) if there was a lick within a 0.15-s time bin or a value of zero (blue) if there were no licks in the time bin. The figure displays the licking behavior of the animal for rewarded odor trials (left) and unrewarded odor trials (right). Blue denotes periods when the mouse was not licking while red denotes licking and ten trials are shown for rewarded or unrewarded odors for each block. Blocks are displayed from top (first) to bottom (last). As shown, the animal learns to respond correctly by refraining from licking in the unrewarded odor trials. Rewarded odor trials in which the animal failed to lick sufficiently were called misses and successful trials were hits. The yellow arrow in the left panel shows a miss (an error that the animal made when it was supposed to lick during presentation of a rewarded trial) within a block where the majority of the responses were correct (hits). Unrewarded odor trials in which the animal licked as if it were the rewarded odor were called false alarms (FA) while trials in which the animal correctly refrained from licking were called correct rejections (CR). The yellow arrow head on the right panel in Figure 5A points to a false alarm in a block where the animal responded with correct rejections during the other unreinforced odor trials. For each unit/odor pair the average firing rate to the unrewarded and rewarded odor during the odor exposure period was normalized from zero to one respectively (Figure 5B). If the licking behavior or the expected outcome was driving the unit responses instead of the odor stimuli then the responses observed during error trials would be expected to resemble correct trials of the other odor. Figure 5B shows histograms compiling the normalized responses in 11 sessions in four animals (these were a subset of the 34 sessions in Figure 4 where the animal made six or more mistakes). The figure shows that for the unrewarded odor on trials when the animal treats it like a rewarded odor (FA) (licking with expectation of reward), the unit still responds with a firing rate that is not different from the correct rejection (CR) (*p* = 0.06, *n* = 30 units). Similarly, while error trials involving the rewarded odor differed between hits and misses (*p* = 0.001, *n* = 23), the normalized response magnitude for misses (Figure 5B) was much closer to one than to zero indicating that the observed unit responses are mainly odor responses and not a response to licking or some feedback representation from higher order areas of expected outcome.

### Divergent SMC Responses Change when the Rewarded and Unrewarded Odors Are Switched

**Figure 6:**
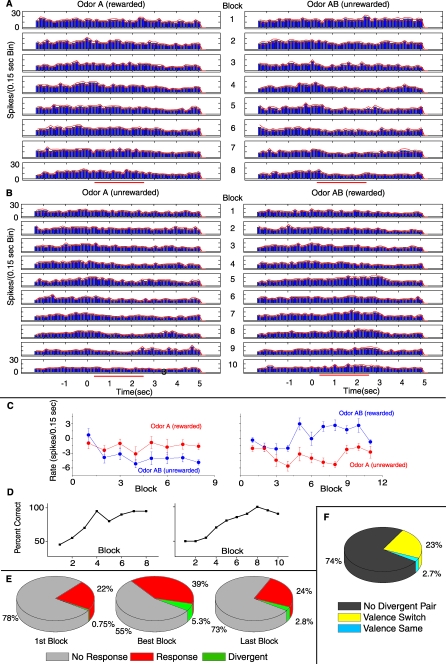
Changes in MC Odor Responses during Reversal (A) PSTH for a multiunit response to odors A and AB during an initial discrimination (day 1) whose behavioral performance curve (percent correct responses versus block number) is displayed in (D) (left). This unit never develops a significant response to A but develops a significant inhibitory response to AB. (B) The same multiunit responding the next day (day 2) in the performance of a reversal task in which the reward associations of odor A and AB had been reversed (the behavioral curve is displayed in the right side of [D]). The unit developed a significant inhibitory response to odor A (now unrewarded) and a significant excitatory response to odor AB (now rewarded). The red bar under the time axis denotes the interval when the mouse was exposed to the odor. (C) Odor-elicited firing-rate changes observed during day 1 (left) and during reversal in day 2 (right). The two plots show unit firing-rate change in response to odor A (red) and odor AB (blue) in each block of the session. The points represent the firing rate in spikes/0.15-s bins during odor exposure (0.5 to 2.5 s) minus the rate in spikes/0.15-s bin in the period before odor exposure (−1 to 0 s) (for details see Materials and Methods). Error bars denote mean +/− SEM of each point (*n* = 10 rewarded and 10 unrewarded odors per block). An ANOVA indicated that in both day 1 and day 2 the responses to odor A differed from responses to odor AB (*p* < 0.05). (D) Percent correct responses for the behavior in the first day task (left) and the second day reversal (right). (E) Summary of all reversal experiments consisting of 358 units from eight animals and 24 different reversal sessions. (F) Pie illustrating what happens to units that are divergent in either the initial discrimination or the reversal. “No Divergent Pair” indicates that the unit only had statistically significant divergence in one of the two complementary tasks. “Valence Switch” means that the odor evoked firing rates switched from A>AB to A<AB or vice versa from the initial discrimination to the reversal. “Valence Same” indicates that the odor evoked firing rates maintain their relationship A>AB to A>AB or vice versa from the initial discrimination to the reversal.

Figure 6A shows the PSTHs for rewarded (odor A, left) and unrewarded (odor AB, right) odors for a multiunit from an animal that has not previously been shown. The left panel in Figure 6C shows the corresponding change in firing rate elicited by the reinforced (red, odor A) and unreinforced (blue, odor AB) odors as a function of the number of blocks, and the left learning curve in Figure 6D shows the corresponding behavioral performance curve. There is no significant change across blocks in response to the rewarded odor (A) but there is development of a reduced firing rate response to the unrewarded odor (AB). Figure 6B displays neural activity recorded from the same electrode as in Figure 6A the following day during a reversal of the reward to odors A and AB, and the corresponding plot in Figure 6C (right) shows the change in firing rate elicited by the odor A (red) that is now unreinforced and odor AB (blue) that is now reinforced as a function of the number of blocks. The plot on the right in Figure 6D shows the corresponding progress through the learning curve as the animal learns the new associations of the odors (A, unrewarded and AB, rewarded; the left plot is the learning curve for the first day A, rewarded and AB, unrewarded). What is interesting is that as the animal learns to make these new associations the unit's response changes polarity. Now in response to the same odor (AB) that resulted in a significant reduced firing rate response on day 1, cells recorded from the same electrode on day 2 developed a significant excitatory response (compare blue curves in the two panels in Figure 6C). It is also important to note that the reduced firing rate response to odor AB gained during the initial discrimination is not present at the beginning of the reversal the following day. This switch was observed for the majority of units that developed divergence for odor responsiveness in the first day and was subsequently tested for reversal the next day (compare the green slice in the first block in Figure 6E with the green slice in the best and last blocks in Figure 4C, Chi^2^ < 0.05). This further illustrates that the observed changes occurring during discriminations are transient, not persisting to the following day.

Figure 6F illustrates how all units involved in a reversal task changed their odor responsiveness and the amount of divergence as the animals learned to reverse the associations learned the previous day to odors A and AB. Despite the animal's preexisting ability to discriminate between odors A and AB, needing only to reassociate the odors with the correct behavioral output, changes continue to occur in the OB. As shown by the pie chart in Figure 6E during the day 2 reversal session SMCs show both a significant increase in the number of responsive and divergent units from the first block to the best block (Chi^2^ *p*-value < 0.05, *n* = 358 units in 24 reversal sessions performed by eight animals) as well as the transient decrease from the best block to the last block (albeit, not significant in a Chi^2^ test). Figure 6F examines whether this valence switch in the divergent response was unique to the unit presented in Figure 6A–6C or whether it is more pervasive. In the animals that performed reversals following an initial discrimination of A versus AB, 74% of the divergent units did not diverge in the complementary task (*n* = 90 divergent units in 18 reversal experiments performed by seven animals). In this case, when the unit diverged in the initial discrimination it failed to diverge in the reversal task or vice versa. The remaining 26% of units had a divergent response in both the initial discrimination and the reversal and 89.5% of those underwent a valence switch as illustrated in Figure 6A and 6B. We observed such behavior in 14 multiunits and three single units. While it is not possible to prove that the same unit was being recorded on both days, it is remarkable that the same cell or other cells in the vicinity of the same electrode showed a divergent response upon reversal. This means that of all the units that yielded a divergent response to odors A and AB in both the initial discrimination and the reversal, 89.5% underwent a valence switch.

### Development of Divergence in SMU Responses Does Not Take Place When the Element of Surprise in the Introduction of New Odors is Abolished in the Odor Discrimination Task

**Figure 7:**
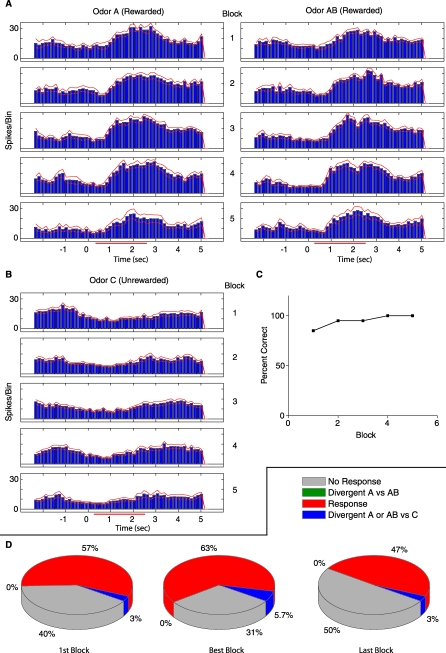
Responses of SMCs to Novel Rewarded Odors A and AB in the Multiple S+ Control Task Do Not Diverge between A and AB (A) PSTH of a multiunit response to odor A (left) and AB (right) both of which are rewarded. This multiunit responds to both odors A and AB with a significant excitatory response. (B) PSTH of the same unit responding to the unrewarded odor C (cumin aldehyde) that the animals were familiar with as an unreinforced stimulus prior to start of the task. This unit displays a significant inhibitory response to odor C. There are twice as many trials of odor C compared to A and AB per block because the ratio of rewarded to unrewarded odors remains at 50% in this task. For every ten trials of odor A or Odor AB there are 20 trials of odor C. (C) Behavior plot corresponding to the raster shown in (A) and (B). It should be noted that the animal performs at criterion from the first block because although odors A and AB are novel, odor C has an overtrained association as the unrewarded stimulus. (D) Summary of all experiments consisting of 200 units from six animals and 12 different task sessions. It should be recognized that the green pie denoting units divergent in A versus AB responses do not exist in this behavior. The added blue pie piece signifies the percent of total units that had a divergent response between either A or AB and odor C. This value did not significantly vary through the course of the task (first, best, and last).

In order to determine whether the observed changes in SMC firing in the OB were the result of olfactory discrimination learning or merely the result of repeated exposure to novel odors paired with reward, an alternative behavioral task was used. In this task the animals were overtrained with the odor cumin aldehyde always predicting the unrewarded trials and any other presented odor predicted a rewarded trial (see Materials and Methods under multiple S+ control task). Two novel odors were chosen based on activation of recorded units as in the previous behavioral task except this time both A and AB were rewarded and the overtrained unrewarded odor, cumin aldehyde, was the S−. The animals no longer had to discriminate between odors A and AB since they had the same valence (rewarded). Odor C (cumin aldehyde) was paired with no reward, an association that the animals had been overtrained to. For this reason they did not need to make any new association between odors and outcomes and this allowed them to perform at criterion immediately (Figure 7C).

Figure 7A displays multiunit activity for odors A (left) and AB (right) both of which were rewarded. Figure 7B shows the same unit's response to the unrewarded odor C (cumin aldehyde). It is obvious from the rasters in Figure 7 that there is a significant excitatory response to odors A and AB and a reduced firing rate response to odor C. Figure 7D illustrates how all unit responses and divergence change through this task. It should be noted that there are no units that display a divergent response to odors A and AB throughout the task, which is in stark contrast to animals learning to discriminate between the two odors (Figure 5D). The blue wedge indicates the percent of cells that show a divergent response between either A or AB and odor C like the unit whose responses are shown in Figure 7A and 7B. The percent of these units does not change significantly from the first block to the best block to the last block (Chi^2^ > 0.05, *n* = 200 units recorded in 12 sessions performed by six animals). It is also interesting that the number of responsive cells does not change significantly through the task either. This task results in a more stable representation of the odors through the duration of the task and illustrates that the observed changes from Figures 3–6 are not simply the result of repeated exposure to the two odors, nor a repeated pairing of those odors with a reward in the absence of an unexpected outcome. This fits with the learning theory of Schultz because it is the unexpected and wrongly predicted outcomes that produce learning-related changes [39].

### Comparison of the Relationship between the Time after Odor Onset When Unit Firing Rates Diverge Compared to the Time When the Animal Makes a Behavioral Decision

**Figure 8:**
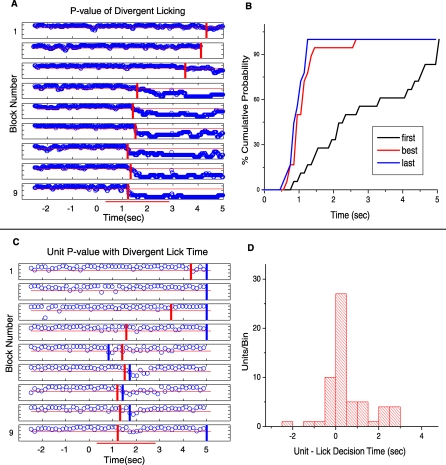
Behavioral Decision and Unit Divergence Times (A) This figure shows the *p*-value of the difference between the lick responses in rewarded and unrewarded trials calculated for each block for the data shown in Figure 5A using a rank sum test at each 0.15-s bin through the trial. As shown in Figure 5A each bin is allocated a value of 1 if the mouse licked at least once or a value of zero if the mouse did not lick. The rank sum test is performed on all zeros and ones in each bin within each block. The vertical red lines indicate when the *p*-value drops below 0.05. The horizontal red lines indicate the 0.05 level. The blocks are shown from top (first block) to bottom (last block). (B) Percent cumulative probability plot summarizing the decision times for the 19 go–no-go experiments where at least one unit diverged when the response to the reinforced and unreinforced odors were compared (the experiments contributing to the green wedge in the pie charts in Figure 4B). The decision time becomes smaller as the animal progresses through the session as evidenced by a shift of the median decision time from 2.5 s for the first block, to 1.0 s for the best block, and 0.95 s for the last block. It is important to state that as explained in the Materials and Methods the actual time of arrival of the odor is delayed ∼300 ms, and therefore what is important in this experiment is not the absolute value of the decision times but rather the relative values. (C) Plot of the *p*-value of the difference between firing rates between odor A trials and odor AB trials compared at different times throughout the trial for the unit shown in Figure 3A. *p*-Values were calculated by using a rank sum test applied to the firing rate calculated in each block in each 0.15-s bin. The horizontal red line is drawn at a *p*-value of 0.05. The vertical blue lines indicate the time at which the difference in firing rate between odor A and AB dropped below 0.05 for two or more consecutive points. Because of multiple comparisons, the *p*-value falls below 0.05 occasionally for a single point as seen by the few single data points that fall below the red line in the period before odor responses (−2 to 0 s). We found that two adjacent data points fall below 0.05 only rarely in this prestimulus period (4% of the time) allowing us to use the criterion of two adjacent points below 0.05 as the time when the two rates differ. The vertical red lines are taken form (B) and allow for a comparison of timing between divergent lick behavior and divergent MC firing. (D) Distribution of the difference between unit divergence times and their corresponding behavioral decision times during the best block. The majority of unit divergence occurs after the animal has begun divergent licking behavior (positive values). Only 20% of the units diverge in their firing rate responses to the rewarded and unrewarded odor prior to behavioral divergence of licking.

Figures 3A and 4A showed examples when an SMC's firing rate diverged between the reinforced and unreinforced odor trials, but how does the time of onset of firing rate divergence compare to the time when the animal makes its decision about the difference in odor identity? In order to examine this question we determined when the licking of the tube that is required for reinforcement with water differed between reinforced and unreinforced odor trials by performing a rank sum test for differences in licking at every 0.15-s bin. Figure 5A shows the licking patterns and Figure 8A illustrates the time course for the *p*-value for the test of lick divergence. The red vertical bars indicate where the *p*-value drops below 0.05 (the time when the licking pattern diverges between unrewarded and rewarded odors). Figure 8B shows the cumulative percent probability for decision times measured in the eight animals in 19 sessions where at least one unit diverged in firing between the two odors. As shown, the decision time becomes quicker as the animal progresses through the session as evidenced by a shift of the median decision time from 2.5 s for the first block, to 1.0 s for the best block, and 0.95 s for the last block. It is important to state that as explained in the Materials and Methods the actual time of arrival of the odor is not at time zero, but is delayed ∼300 ms, and therefore what is important in this experiment is not the absolute value of the decision times but rather the relative values.

Figure 8C shows the *p*-value, calculated using a rank sum test in 0.15-s bins, for the difference in firing rates between the rewarded and unrewarded trials for the data in Figure 3 (*n* = 10 rewarded and 10 unrewarded trials). Unit decision times were assigned to the time when two or more adjacent points fall below 0.05 (indicated by the blue vertical bars, see Figure 3 legend for explanation why this criterion fulfills the requirement of correction for multiple comparisons). In addition to the vertical blue bar we include a vertical red bar that denotes the time point where the *p*-value falls and stays below 0.05 for licks as determined in Figure 8A. A comparison of the timing difference between unit divergence (blue bars) and behavioral divergence (red bars) can be observed for a single unit as shown in Figure 8C. The behavior shown in Figure 8C is typical of the response of a large number of SMCs where the unit divergence time took place earlier only in a few blocks. Figure 8D shows the histogram depicting the distribution of the difference between timing of unit divergence and lick rate divergence for all recorded single or multiunits that showed divergent responses during the best block. For the majority of SMCs that we recorded from (80%), the animal made the odor discrimination (lick divergence) prior to the divergence of the unit's firing rate (positive values in Figure 8D). The function of these late divergent cells could be to help maintain a divergent odor representation that might help maintain a divergent lick pattern throughout the remainder of the required lick period. An alternate hypothesis is that the SMCs would not be contributing to the concurrent decision, but would be facilitating sustained changes in circuits in downstream cortical areas that would then contribute to discrimination in subsequent trials.

### Information Conveyed by All Divergent SMCs Recorded in This Study Can Be Used by an Unbiased Observer for Rapid Odor Discrimination

**Figure 9:**
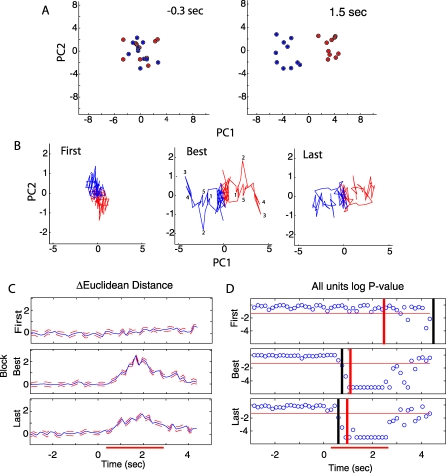
PCA of the Responses of the 95 Divergent SMCs Calculated for the First, Best, and Last Blocks The input to the PCA was the number of spikes per 0.15-s bin for all the trials in each block for the 95 divergent SMCs. The PCA algorithm computes principal components (linear combinations of the input variables) ranked in order of how much of the variance they account for in the dataset; the first few principal components (PC1 and PC2 are shown in [A] and [B]) explain most of the variance and they can be thought of as the firing rate of newly formed units containing a large amount of the information on odor divergence. Note that the principal components are dimensionless and therefore, no units are shown in the graphs. (A) Scatterplots displaying points denoting the location in two-dimensional principal component space of each trial in the best block. The left panel displays the distribution of points at a time before exposure to odor (−0.3 s) and the right panel shows the distribution at a time point during odor exposure (1.5 s). The red points are trials where the animals were exposed to the reinforced odor and the blue points are trials with the unreinforced odor. (B) The entire mean trajectory through time in two -dimensional principal component space is shown for the first (left), best (center), and last (right) blocks. In order to generate the mean trajectory, the mean location of all red and blue points in graphs such as those shown in (A), but spanning the entire time course from −2.5 to 4.5 s was calculated and plotted as a continuous line. Zero seconds is the time when the diverting valve is turned off and the odor is directed towards the animal. The red line is the trajectory for trials with the reinforced odor and the blue line is the trajectory for trials with the unreinforced odor. The trajectories hover around the origin (0.0) during the period before the odor valve opens (It < 0 s) and then move outward in opposite directions after the diverting valve is opened (0 to 2.5 s) during the period when the animal is exposed to the odor. The numbers adjacent to specific points in the trajectory for the best block denote different times in the trial and are shown to facilitate understanding of how the points move through time. The numbers stand for the following times: (1) 0.75 s; (2) 1.05 s; (3) 1.65 s; (4) 2.1 s. (C) The blue line shows the change during the time course of a trial of the mean of all pairwise euclidean distances in PCA space between points that belonged to trials where the animals were exposed to different odors (reinforced versus unreinforced odors). Pairwise euclidean distances are calculated in 95-dimensional principal component space. To calculate the delta distances shown by the blue line the mean of the pairwise distances between points for trials where the animals were stimulated with the same odor were subtracted from the mean of pairwise distances for points for trials where animals were stimulated with different odors. The red traces represent the SEM. (D) Logarithm of the *p*-value calculated with a rank sum test performed at every 0.15-s time bin for the difference in the pairwise PCA distances between points for trials where the animals were stimulated with the same odor compared to pairwise distances for points for trials where animals were stimulated with different odors. If the logarithm of the *p*-value fell below −5, the point was assigned a value of −5. The horizontal red line is the logarithm of 0.05. The vertical black lines are the times when the *p*-value drops below 0.05, and the red vertical lines are the median of the behavioral discrimination times shown in Figure 8B.

As shown in Figure 8D, in about 20% of the SMCs the firing rate diverges during the best block before the animal makes the behavioral decision. If all the divergent SMCs are used, would the information content be enough to allow an unbiased observer to make the decision before the animal has to make the behavioral decision? We used principal component analysis (PCA) to answer this question and to provide lower dimensional representation of how the information content provided by the ensemble of SMCs varied as the animals learned to discriminate rewarded and unrewarded odors. In previous studies PCA has proved to be an effective tool to obtain such information [17,40]. The input to the PCA was the number of spikes fired at 0.15-s intervals for all units that diverged in firing between rewarded and unrewarded odors (95 units from eight animals and 19 sessions, see Materials and Methods). The PCA algorithm computes an orthogonal linear transformation of a dataset into linear combinations of the input variables (principal components) ranked in order of how much of the variance they account for in the dataset; the first few principal components explain most of the variance and they can be thought of as new units containing the majority of the information on odor divergence. Figure 9A displays a scatterplot where the location in a two-dimensional space made up of principal components 1 and 2 is shown for each trial where the animal was exposed to the reinforced odor (red) or unreinforced odor (blue). The plot on the left of Figure 9A is a scatterplot computed at a time before the animal was exposed to the odor. As expected, at this time in the trial the blue and red points overlap and therefore the two principal components contain no information on how to differentiate between odors. In contrast, the panel on the right side in Figure 9A shows the scatterplot at a time point during odor exposure. In this case, there is a segregation of the red and blue points suggesting that there is enough information contained in the two principal components to be able to differentiate between the reinforced and unreinforced odors. Figure 9B shows the trajectory over time for the mean location (calculated over all the trials in each block) for the reinforced (red) and unreinforced (blue) responses plotted in the two-dimensional space defined by principal components 1 and 2. The data are plotted for three blocks: the first and last blocks, and the “best block” defined as the block where units showed the most divergence between odors in each experiment (lowest *p*-values in the odor divergence *t*-test). As shown the blue and red trajectories overlap in the first block, become distinct in the best block, and begin overlapping again in the last block.

The data in Figure 9A and 9B suggest that an unbiased observer may be able to determine the identity of the odor (rewarded versus unrewarded) by using the information contained in the principal components. To evaluate objectively whether this is the case, we characterized the change in Euclidean distance between points in PCA principal component space during the time the odor was introduced. The progression from overlap to divergence and subsequent condensation is illustrated quantitatively in Figure 9C where the Euclidean distance in 95-dimensional principal component space between points belonging to the rewarded and unrewarded odor trials is plotted as a function of time (after subtraction of the distance for points within the same odor group). As shown, the points overlap (mean Euclidean distance is zero) in the first block, they diverge maximally during odor exposure in the best block, and start collapsing together in the last block (difference in Euclidean distances between blocks are statistically significant as determined by ANOVA *p* < 0.05). Interestingly, when the significance of the difference between the Euclidean distances across odors is compared with distances within odors as tested through the time course of the trial using a rank sum test, the patterns diverge quickly and robustly before the median of the behavioral decision (compare black and red vertical lines in Figure 9D). This indicates that combining the information from all divergent units allows for efficient decision making at a time before the animal is making those decisions behaviorally.

## Discussion

MC firing in the OB of awake behaving animals is affected by attention, motivation, and previous experience, and the effects of these contextual variables on firing rate are often of the same magnitude as the sparse putative odor responses recorded from the same cells [33,34,41,42]. Thus, identification of specific odor-induced activity in MCs has been challenging, leading some investigators to question whether the OB in an awake behaving animal is more like cortex as opposed to a primary sensory relay area [43]. In this study we adapt the strategy of screening odor responsiveness in multiple MCs with a relatively large number of odors [35] to identify effective novel stimuli that are used in a subsequent odor discrimination learning session. These experiments are the first to record effectively how the odor responses of SMCs evolve during an olfactory discrimination learning task. We found that both MC odor responsiveness and divergence in differential responses to the two odors being discriminated increased during the discrimination task. The data also showed that the increases in responsiveness and divergence were transient, diminishing by the end of the discrimination task, and gone the following day. Strikingly, when SMC responses diverged while performing an odor discrimination task on the first day, a reversal of the association of the reward with the stimulus on the second day caused a substantial fraction of SMCs recorded from the same electrode to display responses that diverged with the opposite polarity. These results redefine the function of the OB as a transiently modifiable (active) filter modulated by context to shape odor representations at the output of the OB in behaviorally relevant ways.

### What Mechanism(s) Is(Are) Behind the Observed Plasticity

This set of experiments explored the modulation of MC activity during an odor discrimination task. Modulation of MC responses can be effected by centrifugal innervation from higher order centers to the bulb, or by changes in sniff patterns during learning. It is difficult to envision how changes in sniffing could result in divergent responses of MCs to odors in an odor discrimination task and how they would underlie a change in polarity upon switching of the assignment of odor reinforcement. However, changes in sniffing frequency have been shown to alter glomerular activation and so could theoretically result in divergent MC activation patterns [21,44]. Thus, modulation of OB input by changes in sniffing patterns during learning deserves further study.

The transient increase in responsiveness of MCs to the odors being discriminated could be dependent on centrifugal innervation from neuromodulatory centers such as cholinergic, noradrenergic, or serotonergic fibers [4]. Pharmacological blockade of adrenergic input to the bulb affects learning in an odor discrimination task [45] and the transient nature of the changes in MC firing fits with the known transience of noradrenergic locus coreuleus phasic bursting during this type of odor discrimination behavior [46]. This result also makes sense in the context of what is known about noradrenaline action within the bulb, causing both an increase in MC activation [47,48] and alterations in lateral inhibitory strength [24,49]. In fact increases in population responsiveness mediated by noradrenaline have been observed in the hippocampus [50], and block of β-adrenergic receptors alters changes in local field potential elicited by odors on the surface of the OB [30]. Thus, centrifugal modification of OB circuitry is a good candidate for mediation of the observed changes.

The other centrifugal feedback mechanism that could mediate the increased divergent responses is centrifugal modification from olfactory cortex and/or AON. Divergence could be the result of amygdala/orbitofrontal cortex feedback to the bulb through the piriform (olfactory) cortex or the AON. In an appetitive instrumental conditioning paradigm the amygdala/orbitofrontal cortex system is believed to be the critical system for encoding the learned motivational significance of odor cues and utilization of this information to guide behavior [51]. It is known that the piriform cortex receives strong input from the orbitofrontal cortex and the amygdala causing changes in circuit processing [52] resulting in odor representations in piriform that are highly associative [53–55]. It is also known that extensive feedback from the piriform cortex back to the OB exists thereby establishing an indirect connection between the amygdala/orbitofrontal system and the OB [4,55]. It has been shown that neurons of the amygdala/orbitofrontal system develop divergent activity between reinforced versus the unreinforced odors early in training before the animal performs the task correctly similar to results in the piriform cortex and to our results in the OB [56]. Many of the neurons in amygdala and orbitofrontal cortex reverse their selectivity rapidly when the meaning of the odor cues is reversed, which is similar to our findings from SMCs of the OB and those previously reported in the piriform cortex. These studies show that learning induces changes in amygdala before changes in orbitofrontal cortex take place making feedback from amygdala a prime candidate for modulatory feedback of the OB through piriform cortex. The divergence of MC firing in OB could be accomplished through selective modulation of the odor-activated cell population by massive piriform/AON feedback that is thought to gate granule cell activity [22] and could therefore modify lateral inhibition between MCs.

### Modification of MC Odor Responses during Tasks Involving Learning of Novel Odor Discrimination

Several investigators have described changes in OB field potential responses elicited by odors during odor discrimination learning tasks and find that these changes are altered by modification of centrifugal innervation of the bulb [27,30,57,58]. Because changes in field potential measured in the OB are related to either the degree of oscillatory synchronization between neurons, changes in neuronal firing rates, and/or changes in synaptic activity these investigators have postulated that the circuitry within the OB is modified by centrifugal innervation. However, the precise nature of the changes in neuronal firing within the circuitry in the OB remains unknown.

A definitive approach to define the changes in OB during learning is to record from OB neurons during learning. Unfortunately, this tactic has proven particularly difficult because of the sparseness and small magnitude of odor responses [33,34,41,42]. The most thorough previous study of changes in odor responsiveness and the first to report on activity of single OB units during learning in awake behaving vertebrates is that of Kay and Laurent [33]. These investigators concluded that centrifugal innervation modified responsiveness of MCs to odors, but the relatively small number of odor-responsive cells did not allow the authors to make a conclusion about how individual OB units change in odor responsiveness. This data also did not allow for determination of whether the changes in firing rate were a reflection of behavioral events linked with odor responses or direct responses to odors themselves. Finally, in the antennal lobe of insects Daly and coworkers [28] and Yu and coworkers [29] concluded that antennal lobe projection cells undergo dramatic changes in responsiveness during learning, but the functional significance of these changes was not understood. Our study has made a significant leap forward in assessing changes in MC ensemble responsiveness that occur during normal adult olfactory learning because we were able to systematically record from SMCs and show that learning during an odor discrimination task resulted in divergence in odor responses of individual cells. Importantly, data recorded during trials where the animal makes the wrong behavioral decision (Figure 5) allowed us to conclude that the changes in MC responsiveness during learning reflect plasticity in odor responsiveness as opposed to changes in MC firing that are due directly to centrifugal input or to actions, such as licking, which are associated with odor exposure. Thus, during odor discrimination learning more MCs respond divergently thereby facilitating discriminative responses to odor stimuli. It is also possible that divergent MC activity could be contributing to plasticity in networks downstream from MCs leading to better and longer lasting divergent representations of the discriminated odors.

### Comparison with Previous Findings of Long-Term Changes in MC Responsiveness to Odors

Past studies of OB function have shown that certain learning tasks elicit long lasting changes in responsiveness of MCs to odors. In comparisons of MC population responses, Keverne and coworkers have shown that MCs respond to lamb odors more strongly after parturition. These changes in MC responsiveness are thought to be associated with recognition of offspring by the ewe using smell [59]. Similarly, in rat pups Wilson and Leon have shown long-term changes in responsiveness of populations of MCs studied in anesthetized animals before and after early preference learning, a paradigm where the neonatal rat learns to prefer an odor that is paired with stroking [60]. The finding of long-term changes in odor responsiveness of MCs in these studies contrast with our finding of transient changes in MC firing. Why did we observe transient changes in MC responsiveness while these investigators observed long-lasting changes? One explanation is that these are fundamentally different kinds of olfactory behaviors that utilize the OB in substantially different ways. In fact, it has been shown that a very large release of noradrenaline is involved in the olfactory behaviors described by Keverne and coworkers and Wilson and Leon [61–63], while only modest phasic increases in noradrenaline or locus coreuleus neuronal firing are involved in behavioral tasks such as the odor discrimination task used in this study [12,46]. Indeed, in a recent study in mice Shea has found long lasting repression of MC responses to odors paired with robust locus coreuleus stimulation in anesthetized adult animals [64]. This implies that the intracellular second messenger mechanisms activated by noradrenaline can result in long lasting changes in adult OB. The strength of locus coreuleus stimulation used in the Shea study is likely to result in release of large amounts of noradrenaline greatly exceeding the phasic release that occurs in an adult awake behaving animal during odor discrimination learning [46,62]. Thus, Shea's locus coreuleus stimulation protocol more closely mimics the behavioral situations used by the groups of Leon and Keverne [59,60]. This difference, as well as other differences in the involvement of different centrifugal pathways innervating the OB could explain why permanent changes to MC responsiveness are observed by these investigators and not in our study.

### Conclusion

The earliest stage of processing of olfactory stimuli, the OB, is directly connected to the amygdala and entorhinal cortex, fundamental structures of motivation and memory, and which is unique compared with the early sensory relay centers from other sensory modalities [4,25,43]. Information from the OB does not relay through thalamus, unlike other sensory systems and outputs directly to olfactory cortex, a three-layered paleocortex considered by some to be ancestral to the six-layered neocortex that processes the other sensory modalities. The OB is also uniquely tied to many of the fundamental neuromodulatory centers of the brain as well as receiving robust central feedback from higher order olfactory centers. We show in this study that at the level of the MC the early neural representation of odor is shaped by previously derived meaning, theoretically allowing for more efficient sampling of odor space and providing a simplified yet biased interpretation of incoming stimuli. This subjective modulation of the representation of odor information at the MC layer would endow the system with efficient sampling and readout of this complex odor space. The processed output from the OB would facilitate efficient multimodal associative processing in olfactory cortex. Our study places the olfactory system on the subjective end of the continuum of subjective versus objective early sensory representation thereby providing a new framework for future understanding of early odor signal processing.

## Materials and Methods

### Surgery for implantation of microarrays.

Eight animals were implanted bilaterally with 4 × 2 electrode arrays. Two issues that we judged were important to ensure success in surveying sparse responses of MCs were consistent placement of the array in the same place in the bulb and mechanical stability afforded by using long electrode shanks that would target the ventral MC layer. Surgery for bilateral implantation of electrode arrays was performed on 8–10- wk-old C57BL/6 mice. Animals were anesthetized with an intraperitoneal ketamine xylazine injection. The electrode arrays were manufactured by Micro Probe Inc. and were constructed of platinum iridium wire etched to a 2-μm tip and coated with parylene C to an impedance between 3 and 4 MΩ at 1 kHz. The arrays were organized in a 2 × 4 pattern with 200-μm spacing with lengths of 3.2 to 3.8-mm angled at 45 ° along the long axis. Angling of the arrays ensured optimal targeting of the MC layer that runs at a 45 ° angle with respect to the dorsal surface. The arrays were implanted 0.8 mm anterior to the supra-orbital vein and 1 mm lateral to the saggital suture, and the electrodes were lowered to an average depth of 2.5 mm targeting the ventral MC layer. Recording from all electrodes was performed during implantation to ensure proper placement within the ventral MC layer.

As reported by Kay and Laurent [33] and Rinberg and coworkers [34], with the electrodes used in this study, no spikes were detected while the electrodes traversed the granule cell layer. Once the electrode reached the ventral MC layer spikes with amplitudes ranging from 50 to 500 μV were detected. Once the MC layer was reached, the arrays were fixed in place with titanium skull screws and nail acrylic with one of the titanium screws and a silver wire from one of the arrays serving as the ground. Because these units are recorded at the level of the MC layer, we term the recorded units “suspected mitral cells” (SMC). All animal procedures were performed under protocol approved by the institutional animal care and use committee of the University of Colorado Denver.

### Overview of training and behavioral tasks.

After animals recovered from surgery (∼2 wk) behavioral training began following water deprivation to 80%–85% of predeprivation body weight. Before the animals underwent awake-behaving recording they were trained on two complementary tasks (the odor discrimination task and the multiple S+ control task) that are described below in detail. In the odor discrimination task the animals learned to lick a tube when a reinforced (S+) odor was present and not lick on the tube when the unreinforced odor (S−) was present. The mouse was rewarded with water for licking on the tube on the S+ odor. In the complementary multiple S+ control task, the same paradigm was used, with the notable exception that multiple odors were presented as the S+ reinforced odor while the distinctive odorant cumin aldehyde (1% in mineral oil, odor C) was presented as the S− odor. Because many different odors were presented as S+ the mouse learned to cue on odor C (the cumin aldehyde S−) in the control task.

Once the mice were trained in both tasks awake behaving recording sessions began. On the first day mice were screened with a variety of odors for single unit or multiunit responses using methods outlined below. Overnight we analyzed the data and determined which odors elicited responses. Two of the odors the mice were responsive to (A and B) were chosen for the next day and the mice were run on either the odor discrimination task or the multiple S+ control task (see Table 3 for odors used). For those mice undergoing the odor discrimination task on the second day we used A as the S+ and a 50% mixture of A and B (odor AB) as the S−. The third day the mice were run on the odor discrimination task with a reversal of odor presentation: AB as S+ and A as S-. Finally, other animals were run on the second day on the multiple S+ control task with odors A and AB as S+ and odor C as S−. Notice that the mechanics of the odor discrimination and the multiple S+ control task are quite similar. In addition, odors A and AB have never been presented in either of these tasks to the animals. However, there is a critical difference between these tasks. In the odor discrimination task both S+ and S− (A and AB) are novel odors in the task and the animal must learn to discriminate among them to obtain reward. In contrast in the multiple S+ control task, while the odors A and AB are novel, odor C is an odor that the mouse knows to cue on. Because of this, the control task looses its element of surprise and the animal does not have to learn the task as it ignores odors A and AB, and it cues on odor C to guide acquisition of reinforcement immediately. Thus, the differences between the odor discrimination and multiple S+ control task are that the mouse engages in associative learning in the former, but not in the latter.

**Table 3:**
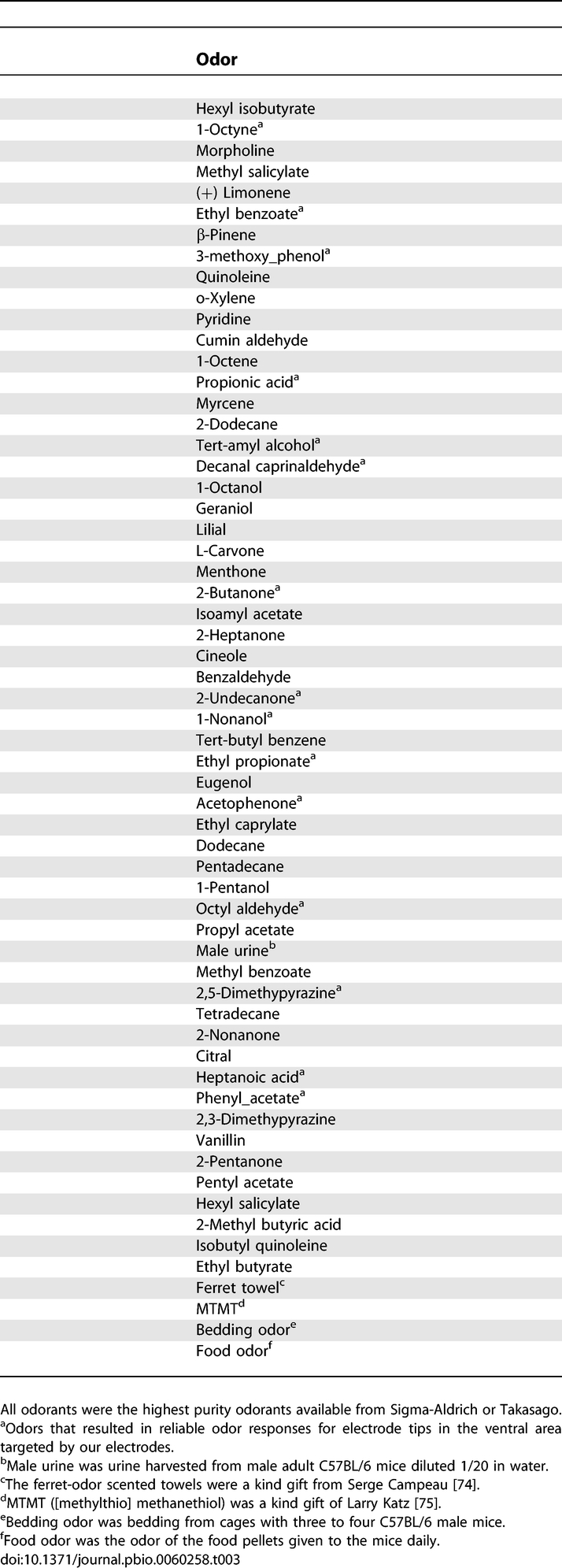
List of 60 Odors Used in Odor Screening

### Odor discrimination test.

For the odor discrimination test the olfactometer and instrumental conditioning methods described by Slotnick and Restrepo [37] were used. Briefly, the mice were trained using water reinforcement. The mice initiated the trial by poking into the odor port. From 1 to 1.5 s after the mice poked into the odor port, they were asked to sample a 2.5-s stimulus presentation and respond by licking a water delivery tube at least once in the last four 0.5-s periods in the presence of a rewarded (S+) odor and to inhibit responding in the presence of an unrewarded (S−) odor (these are the go–no-go reward criteria). After the trial, there was a 6-s timeout during which the animal was unable to initiate a new trial. After this timeout period the animal was able to initiate a new trial at will resulting in intertrial intervals that were at least 6 s in duration. All mice were trained on 1% isoamyl acetate versus 1% cumin aldehyde (v/v in mineral oil) in this automated go–no-go odor discrimination task. Water-deprived mice were trained to distinguish the water-rewarded odor (isoamyl acetate, S+) from the unrewarded odor (cumin aldehyde, S−) by their licking response. All animals were able to discriminate between isoamyl acetate and cumin aldehyde (unpublished data). The animal's performance was evaluated in blocks of 20 trials (ten S+ and ten S− trials presented at random). Each block's percent correct value represents the percent of trials in which the odors were identified correctly. The initial training served to acquaint the animals with procedures in the go−no-go task. Once animals learned to discriminate isoamyl acetate from cumin aldehyde, they were trained on a multiple S+ control task described below. Each session included at least six, but no more than ten blocks of 20 trials.

When the mouse poked its nose into the odor chamber a diverting valve was turned on routing the air flow to room exhaust and away from the chamber and the odor valve was opened. As explained in Slotnick and Restrepo [37], this serves to make the onset of odor delivery into the chamber abrupt. The diverting valve that starts delivery of the odor to the odor sampling chamber switches 1–1.5 s later (at time 0), but delivery of the odor to the animal is delayed because the valve is connected to the odor sampling chamber by a 30.48-cm-long delivery tube with 0.317 cm in diameter that delivers the air at 2 l/min. The delivery tube is attached to the bottom of the chamber where we placed a set of plastic corkscrew mixers (Cole Parmer). While the use of the corkscrew mixers ensures well-mixed mixtures are delivered to the animal, this results in a delay of odor delivery that we estimate at ∼300 ms.

### Multiple S+ control task.

Following training on the two odor discrimination task mice were then trained to be able to perform an additional task. In the new task there were multiple odors that were rewarded (S+) but only one odor (1% cumin aldehyde in mineral oil) that was unrewarded (S−). The ratio of rewarded to unrewarded trials remained at 50%, the same as the two odor discrimination except that up to six odors were chosen at random to be the rewarded odor. Animals learned to refrain from licking on the unrewarded odor (cumin aldehyde) and to lick on any odor that was not cumin aldehyde. This allowed us to rotate any odor into this behavioral task where the animal would perform correctly without any learning. After animals were trained in both tasks they were capable of performing either at any given time because the ability to perform the odor discrimination did not interfere with the ability to perform the control task (and vice versa).

### Odor screening.

Given the specificity with which olfactory receptor neurons innervate MCs and the large number of different receptors (∼1,000) we did not know apriori what odor stimuli would be appropriate for use in our studies. Previous recordings from awake animals have revealed the sparseness of MC activation by randomly selected odor stimuli [34,35]. We decided to screen each animal with 60 odors spanning functional groups as well as carbon chain lengths and configurations (Table 3). The choice of targeting the ventral MC layer was to ensure the maximum mechanical stability for the electrodes. Because all of our electrodes were placed in the ventral MC layer we included odors known to stimulate ventral glomeruli in rat and mice [6,65,66], yet in order not to bias our search we did not limit odors just to those known to target the ventral bulb. A large fraction of the information was obtained from the glomerular archive of Michael Leon and Bret Johnson (http://leonserver.bio.uci.edu/) as well as from information obtained directly from them on odor responses in the mouse ventral glomeruli.

In order to screen these odors in a behaviorally neutral setting an 8 × 8 × 13-cm chamber was constructed in which the mouse could rest and passively be exposed to odors utilizing the odor delivery system of our olfactometer. Odors were introduced on a constant background odor stream for 2 s with an intertrial interval of 60 s. Odors were racked four or five at a time and presented in random order until five trials of each odor had been attained and then new odorants were racked and screened in the same manner. Odors were screened in groups of 12 or 15 per session. After a session the data were analyzed overnight and the best two odors were chosen to be used in an odor discrimination task in which the animal learned to discriminate one of the odors (A) from the mixture of the two odors (AB). Table 3 lists all the odors used in this study. Those shown in red were found to elicit responses more often than the others.

### Odor pair selection.

As in our previous study, in order to make the odor discrimination task more difficult, we asked mice to discriminate between odor mixtures [45]. Odor mixtures have been employed in several recent studies of the speed of olfactory processing [67,68] and odor similarity determinations [45,69]. In our behavioral paradigm the animals learned to discriminate between odor A and a 1:1 mixture of odor A:odor B at an overall concentration of 1% by volume in mineral oil. Importantly, estimation of air odor concentrations [37,70] indicates that odor concentrations were well below trigeminal thresholds for mice [71]. Odor concentrations were at least ten times greater than detection thresholds indicating that the odor pairs were not at perithreshold concentrations. It is important to note that our olfactory task does not differentiate between discrimination based on intensity, quality, or both.

### Recording setup.

In order to bridge the two electrode array outputs into a single headstage input a small adapter bundle was used containing two straight nine-pin omnetics connectors at one end that interfaced with the electrode arrays and the ground screw and an 18-pin omnetics at the other end that plugged into a 16-channel TDT headstage (TDT: Tucker Davis Technology). The 1× gain headstage was connected by a short cable to a TDT motorized commutator that was connected to a custom-built reference selector box before input to a CWE 16-channel amplifier and bandpass filter. The reference selector allowed us to choose what electrode in each array would serve as the reference for the other electrodes for differential amplification. The signal from 14 electrodes was amplified 2,000 times and filtered at 300–3,000 Hz before outputting to a Data Translation Inc DT3010 A/D card in a PC. The two channels acting as reference for the other 14 channels were amplified in single-ended mode using the screw on the animal as the reference at a gain of 500× and filtered at 1–100 Hz for local field potential recording. Data acquisition was controlled with custom software written in MATLAB (Mathworks, Inc.) and acquired data from each electrode at 24,000 Hz with a gain range of +/− 1.5 volts. Digitized behavioral events output by the Slotnick olfactometer (licks, nose pokes, and odor on) were also acquired in real time with the Data Translation card and recorded by MATLAB onto the PC hard drive. The data were recorded in continuous 9-s streams that encompassed each trial. All analysis was done offline after the completion of a recording session.

### Offline analysis.

Custom software written in MATLAB was used to threshold each channel at 3× RMS of the baseline noise. Every thresholded spike (24 points at 24 kHz) was then saved from each channel and imported into a second program where we clustered the waveforms of similar shape by performing wavelet decomposition and superparamagnetic clustering using the method and MATLAB software developed by Quiroga and coworkers [36]. We made a minor modification to the software. In addition to determining 18 wavelet coefficients used in the Quiroga program, our modified program also determined the first three coefficients of a PCA of the spikes and calculated the peak to valley ratio. As explained in Quiroga et al.[36], the program then proceeded to determine which of these descriptors showed a multimodal distribution and used the ten best descriptors to separate the spikes into well defined clusters using superparamagnetic clustering. Figure 1C shows one example of which descriptors were chosen for analysis. The upper panels in Figure 1D show the separation of waveforms for this example and the lower panels in the same figure show the ISI histograms for the two clusters. We defined a single unit using the criterion of finding <3% of the spikes in the refractory period of 2 ms in the ISI histogram. Figure 1D shows one single unit (left) and a multiunit (right) defined using this criterion. On the average we obtained 12 multiunits and five single units per experiment. Waveform clusters that were considered to have originated from the same neuron (<3% with an ISI less than 2 ms), displayed overall firing rates between 5 and 35 Hz. We examined the stability of the classification method over time to ensure that single units were not misclassified at different times in the task. This was done by ensuring that the factors (wavelet coefficient, PCA components, or peak to valley) used in sorting the data did not change systematically as a function of time across the session.

### Data analysis.

Unless otherwise noted all data analysis was performed using custom written MATLAB programs.

### Sliding window *t*-test to classify units as “divergent.”

Within each block differences between firing rates in response to the different odors during odor application (ten rewarded odor trials and ten unrewarded odor trials) were assessed using a *t*-test for the firing rate calculated in 0.75-s windows that were slid by 0.375 s to span the entire interval between 0.5 to 3.125 s (the peristimulus interval). Thus, for S+ and S− firing rates were compared in 0.75-s windows with their leading edges located at 0.5, 0.875, 1.25, 1.625, 2, and 2.375. These comparisons were performed within each experiment in each block for every unit resulting in multiple comparisons. Within each experiment, the calculated *p*-values were corrected for multiple comparisons using the false discovery rate (FDR) method [72]. The difference in response between different odors was judged significant when the *p*-value fell below the FDR *p*-value in at least one 0.75-s window in the peristimulus interval.

As a control, differences in firing rate in the same block were compared between rewarded and unrewarded trials using the same procedure in the interval from −2.5 s to 0 s in the absence of odor (the prestimulus interval) to assess the effectiveness of the correction for multiple comparisons. Occasionally a single block was found to be significantly different between rewarded and unrewarded trials in the reference interval, but the firing rates were found to be significantly different in the reference interval in two or more blocks in only one of 660 units (compare to 95 of 660 units in the peristimulus interval). Accordingly, we adopted the conservative measure of classifying a unit as divergent only when the *p*-value for the *t*-test of significance of differences between firing rate in the odor exposure interval was below the FDR-corrected *p*-value in at least one 0.75-s peristimulus window in two or more blocks.

### Sliding window *t*-test to classify units as “responsive.”

In a separate set of *t*-tests, the rate of firing in the odor (peristimulus) interval was compared to the firing rate during the prestimulus interval using a similar procedure and correction for multiple comparisons with the exception that the firing rate in the reference interval (from −1 to 0) was compared to the firing rate in 0.75-s windows that were slid by 0.375 s to span the entire interval between 0.5 to 3.125 s (the peristimulus interval). A reference interval of −1 to 0 s was used in this task because occasionally there are contextual changes in firing rate in the interval between −2.5 to −1 when the mouse is preparing to poke its nose into the sampling chamber. As in the case of comparison of firing rate between odors described above, the FDR was used to correct for multiple comparisons, and a unit was classified as responsive only if *p*-values fell below FDR in at least one 0.75-s peristimulus window for two or more blocks.

Compared to more conventional methods (e.g., testing the difference in a single peristimulus interval), the use of a sliding window and correction for multiple comparisons using FDR allows finding differences in units whose responses peak at different times. While to our knowledge FDR has not been used in comparison of unit behavior in multielectrode array recordings, it is known to be a robust well-grounded statistical method and it is used routinely in gene chip analysis and other multiple comparison tests [72]. The use of a more conventional method (using single pre- and peristimulus intervals) yielded qualitatively similar results for comparisons of SMU behavior (unpublished data).

###  PCA.

PCA was computed using the princomp function in MATLAB. The input was the firing rate computed in 0.15-s bins through the time course of the odor response for ten rewarded and ten unrewarded trials for all units that showed divergence (as determined statistically as described above). A total of 95 units from eight animals and 19 experiments where at least one unit diverged in responses to the two odors were included in this analysis. Before input to the PCA, the firing rates were converted to a z-score using MATLAB's z-score function.

## Abbreviations

AON: anterior olfactory nucleus
FDR: false discovery rate
ISI: interspike interval
MC: mitral cell
OB: olfactory bulb
PCA: principal component analysis
PSTH: peristimulus histogram
SD: standard deviation
SEM: standard error of the mean
SMC: suspected mitral cell
